# The linguistic and emotional effects of weather on UK social media users

**DOI:** 10.1038/s41598-024-82384-w

**Published:** 2025-03-07

**Authors:** James C. Young, Rudy Arthur, Hywel T. P. Williams

**Affiliations:** Computer Science, Innovation Centre, University of Exeter, North Park Road, Exeter, EX4 4RN UK

**Keywords:** Computational science, Environmental impact, Environmental health, Psychology and behaviour

## Abstract

Weather significantly impacts mood and happiness, yet observing this at scale and differentiating across weather types is challenging. This study examines the variation in public sentiment related to different weather conditions, as reflected in the vocabulary used in UK-based social media (Twitter) content. We introduce a novel context-sensitive sentiment metric to construct scales that rank words and emojis by both weather severity and emotional intensity, controlling for linguistic variations that naturally occur in different discussion topics. Our findings reveal that emotional responses to weather are complex, influenced by combinations of weather variables and regional language differences. For five weather conditions (temperature, precipitation, humidity, wind speed and barometric pressure) we first identify the sentiment and weather severity associated with words commonly used to discuss them, highlighting the distinct vocabulary used to express positive and negative emotions for each weather type. Next, we demonstrate that language used in weather discussions predicts the severity of each condition and varies across different weather combinations. These findings highlight the importance of context-sensitive sentiment methods for better understanding public mood in response to weather. This approach reveals systematic relationships between weather conditions and public mood, offering insights for impact-based weather forecasting and risk communication.

## Introduction

The effect of weather on mood and subjective well-being has long been recognised^1^. This applies to both transient weather conditions^2–5^ and the overall climate^6,7^. Social media, particularly Twitter, has been key to understanding this effect in the past decade. Research conclusively demonstrates that social media users discuss weather and weather events^8–11^ and that weather affects the sentiment of these discussions^12^. This applies to acute weather events like storms, hurricanes, and heatwaves^13–15^, as well as general trends, such as the tendency for sentiment to decrease with rising humidity^16,17^.

Understanding weather-related mental health effects is crucial in light of climate change. Research finds that increasing temperatures are associated with negative mental and physical health^18,19^. The idea of Shifting Baseline Syndrome^20^, a gradual change in what is accepted as normal^21^, applies here. Moore et al.^22^ show that the notability of extreme temperatures is decreasing over time, with the socially accepted idea of “normal weather” adjusting on a 5-year timescale. At the same time, they also find this is not accompanied by a decrease in negative sentiment, implying no adaptation is occurring. The lack of adaptation to increasing temperatures is confirmed with other measures of mental health outcomes, like suicide rate and number of emergency department visits^23^.

Climate change is not only increasing temperature but also increasing prevalence of extreme and volatile weather. Social media analysis suggests that the shifting baseline effect—where people become less likely to notice changes in ambient weather conditions over time if there are no accompanying harm reduction measures (such as increased access to air conditioning, heatwave warning systems, or urban planning adaptations)—also applies to various types of weather. For example, Weaver et al.^24^ find that the same absolute wind speed is reported online as stronger or weaker depending on the typical weather conditions in the local area. Zhang et al.^25^ find that extreme weather events decrease in notability as they increase in frequency. Thus, a proper understanding of the public response to weather using social media data necessitates methods that can account for different baselines across time and space. That is, the study of the emotional impact of weather requires sentiment analysis methods that are context-sensitive and thereby able to control for the shifting baseline effect.

As well as accounting for physical and climate factors, there remain a number of technical challenges in understanding weather and climate perception from social media data. Typically, the emotional valence of social media posts (most often tweets) is measured using sentiment analysis algorithms applied to text content^26^. Research using such methods to understand social media related to weather and climate increasingly finds that higher accuracy requires a better understanding of the context of the discussion^27,28^. For example, words such as “active”, “erupted” and “fiery” have very different meanings in the context of a volcanic crisis than their more common uses in general conversation^29^. The term “global warming” is used particularly by those who deny its seriousness or urgency, as opposed to terms like “climate change”, “climate crisis”, or “global heating”, which are more commonly used by those emphasising the severity of the issue^30,31^. This difference in terminology reflects varying perceptions and attitudes toward the subject. Sentiment analysis can also be confounded by regional lexical variations in social media content^32,33^. For example, Grieve et al.^33^ find that the term “pissed off” is much more common in tweets from England than in tweets from Scotland, whilst the opposite is true for the word “angry”. Methods that used fixed sentiment valence scores for a given word, ignoring the particular context in which the word appears, are likely biased by failing to account for topic-dependent and geographical variation in usage.

While large language models like ChatGPT^34^ and BERT^35^ usually achieve high accuracy on general natural language processing benchmarks, they can suffer from hidden biases^36^ and still struggle with dialectal and non-standard English^37^. Transfer learning approaches can retrain large language models for specific contexts, however, such efforts can be computationally expensive and require significant amounts of training data^38^. These are also “black box” models which lack explainability^39^. When informing safety-critical decisions, like sending evacuation alerts or weather warnings, being able to explain why decisions are made is crucial for institutional trust and compliance^40^.

Therefore, there is a need for sentiment analysis methods that are both context-sensitive and transparent. This paper addresses this gap by employing “white box” techniques that account for weather-domain-specific language and regional lexical variations, enabling a more accurate and explainable analysis of the relationship between weather and public mood.

This paper uses a context-sensitive sentiment analysis technique to evaluate the impact of weather conditions on public mood, using a large collection of social media (Twitter) content from the UK. In doing so, it seeks to control for cultural, geographic and climatic baselines by constructing sentiment scales specific to the local context. The aims of this study are to:

1. Normalize weather responses to account for local conditions, allowing the effects of absolute versus relative anomalies to be disentangled; this is important for determining whether public mood is influenced more by actual weather conditions or by deviations from typical local weather.
2. Apply context-sensitive methods to understand the relationship between weather and public mood, accounting for: (a) Weather-domain specific language; and (b) Regional lexical variation.
3. Use “white box” methods, so that results are explainable.
4. Study the effect of combinations of multiple weather conditions on public mood e.g. high temperature with high humidity versus high temperature with low humidity.

The structure of the paper is as follows: Sect. “Methods” details the data collection and filtering processes for both Twitter and weather data, as well as the normalisation and context-sensitive sentiment methods. Section “Results” presents the findings from the analysis. Section “Discussion” interprets these findings, discusses their implications, and suggests avenues for future research.

## Methods

### Twitter data collection

The tweets used for this study were collected using the academic Twitter Application Programming Interface (API) V2, which has since been deprecated. Since we are researching human responses to weather, it was necessary to filter out automated tweets. We chose to study content from the UK as a case study where there is substantial geographic variation in dialect and climatic conditions. The following filtering steps were taken:

1. *Thematic collection* The initial dataset contains all tweets from 2021 with the term “weather” in their text (case insensitive), resulting in 8,175,136 unique tweets as identified by their tweet IDs.
2. *Geolocation* Our focus is on tweets originating from the UK. Twitter allows users to geotag their tweets with a specific location, which returns a GeoJSON bounding box, or have their location settings enabled, which returns a GeoJSON point. Given that fewer than 1% of tweets are geotagged using these methods^41^, location inference was necessary for the majority of the tweets. Following the methodology used by Arthur et al.^9^, which builds on Schulz et al.^42^, various indicators were cross-referenced (such as tweet text, user descriptions, and user-provided locations) against gazetteers (GADM, DBpedia and Geonames^43–45^) to infer the tweet’s predicted location through the most probable overlapping polygon. This process identified 1,099,124 tweets as originating from the UK.
3. *Bot account removal* In this context, bots are defined as accounts that produce automated, non-human generated tweets. A simple removal method is employed here, which involves removing tweets from accounts that individually contributed to over 1% of all tweets in the dataset (as previously utilised by Arthur et al.^9^). This process removed five accounts (“BodatHome ”, “Favershamweather”, “Northampton Weather”, “Rayne Weather”, “Sigginstone Weather”), which upon manual inspection showed a high percentage of automated weather updates. After this removal, the dataset contained 1,012,319 tweets.
4. *Weather account removal* Whilst the above method removed the high tweeting accounts, many smaller accounts remained in the dataset that posted high volumes of unwanted, automated tweets. These were predominantly local weather accounts providing structured updates on their regional conditions, rather than the desired, human-produced weather content. All tweets from accounts with “weather” in the username were removed, identifying 1325 unique accounts. A manual inspection of these accounts and their tweets showed a high accuracy of this filter. This reduced the dataset to 487,151 tweets.
5. *Text filter* An inspection of 200 tweets showed a high percentage (18%) of tweets that passed the above filters still contained automated weather content. These tweets were highly structured, containing a variety of weather conditions listed. They could be identified and separated through their presence of the meteorological terms mph (miles per hour) when discussing wind speed, or hPa (hectopascal) when discussing atmospheric pressure. Therefore, all tweets containing mph or hPa (case insensitive), unless found within a word (e.g. oomph or toothpaste) were eliminated. Additionally, tweets containing the phrase “under the weather” were also removed, as this accounted for approximately 1% of weather tweets. This final filter reduced the dataset to 401,160 tweets.

A manual inspection of 200 random tweets showed a high relevancy of 98% which sufficed for this investigation.

### Weather condition data collection

The weather conditions investigated for this study are maximum daily values for temperature, wind speed, precipitation, humidity, and pressure. These conditions were selected because they are key indicators of weather patterns and have significant impacts on human comfort, health, and behaviour, making them crucial for understanding how weather influences public sentiment.

The weather data for this research was obtained from the E-OBS dataset, which provides a daily gridded land-only observational dataset over Europe^46^. This dataset is available on a grid with a spatial resolution of 0.25°. Although a higher resolution of 0.1° was available, the 0.25° resolution sufficed for our purposes as it was finer than the majority of the polygons obtained through location inference. After filtering to retain only grid points covering the UK, we obtained 574 gridded observations per day, each containing 5 different weather measurements. This dataset achieves an average daily coverage of 98.2% across all weather conditions, accounting for occasional data dropout on some days.

Each tweet in our dataset that was successfully geolocated and filtered was then cross-referenced with the E-OBS gridded weather datasets, aligning them by time and geographical coordinates. For tweet geometries overlapping multiple grid points, the condition values were averaged; for those between points, the conditions at the nearest point were used. This process assigned every tweet in the dataset five weather conditions, estimating what the user experienced at the time and location of the posted tweet.

To adjust for regional variations in weather conditions, recognising, for example, that 25 °C is more common in London than in Inverness, we calculated z-scores for the weather conditions associated with each tweet. These scores normalise the data and facilitate comparisons across different regions. The z-score for each tweet’s weather condition was calculated using the formula:

$$\begin{aligned} z_C(x,t) = \frac{C(x,t) - \mu _C(x)}{\sigma _C(x)} \end{aligned}$$

In this equation, $z_C(x,t)$ represents the z-score for the weather condition *C* at location *x* and time *t*. *C*(*x*, *t*) denotes the observed weather condition (temperature, pressure etc.) at the tweet’s location, $\mu _C(x)$ is the average weather condition for that location over the previous 10 years (2011-2020), and $\sigma _C(x)$ is the standard deviation of the weather conditions at that location over the same period. This method ensures that the weather conditions associated with each tweet are evaluated relative to the historical weather variability of its specific location, thus normalising the data for more reliable regional comparisons.

### Linguistic analysis

We used the Python library CIDER^47^ to analyse the language used within tweets. The reader is referred to the paper for the detailed discussion of this algorithm, see also^48,49^. Briefly, this library performs domain-specific linguistic analysis by taking a text corpus and two sets of oppositely polarised seed words as inputs. CIDER then generates a custom dictionary based on the corpus which can be used to classify text. For example, to use CIDER for sentiment analysis a positive-to-negative scale is created by providing sets of positive and negative seed words e.g. {excellent, joy, ...}, {terrible, misery, ...}. The algorithm then uses a network of word associations derived from the whole corpus to discover first, second and higher relationships between the seed and other words. The output is a valence dictionary e.g. {good:0.7, bad:-0.6, average:0.05, ...} that is customised to the specific corpus under study. This custom dictionary is then used, together with modifiers accounting for grammatical features of the text like negation, emphasis etc.^50^, to score sentences (or, in this case, tweets). Since CIDER works with linguistic tokens rather than words specifically, it will also generate valence scores for any emojis present in the corpus.

CIDER can also be used to create scales other than sentiment, such as hot-to-cold, north-to-south, or male-to-female, by selecting seed words associated with the extremes of these dimensions e.g. the term “ice-cream” is commonly associated with high temperatures, so would be a hot word, while “snowman” would be on the opposite end of the hot-to-cold spectrum. In such cases, the valence scores assigned to words/emojis represent positions along the defined dimension, e.g. scores for “hotness” or “coldness”.

The classifier built by CIDER is accurate, lightweight, and explainable and has been shown to be the best in the class of dictionary based sentiment analysis methods^47^. While it is outperformed for sentiment analysis in terms of raw accuracy by LLMs, for understanding and synthesising a large text corpus we trade accuracy for explainability. The resulting word-level polarities can be viewed to provide context at the aggregate level and the reason for individual tweets achieving their scores can be readily determined, which would be difficult to achieve with LLMs or “black box” methods.

#### Sentiment analysis

Sentiment analysis is a natural language processing (NLP) methodology that quantitatively summarises the emotion in text. For instance, “I love the weather” may be assigned a score of +1 (positive), whilst “This weather is horrid” may be assigned a score of $-1$ (negative). For this study, we trained CIDER to assess sentiment within the context of our filtered weather tweet corpus. The trained CIDER classifier was then used to measure sentiment for individual tweets, providing sentiment scores between $-1$ and +1 that reflect the emotional tone of the content. The seed words selected to create this spectrum are the same as those used in the original CIDER paper^47^:
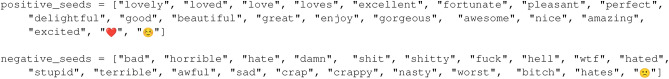


#### Weather condition lexicon analysis

CIDER was separately trained to create dictionaries that assign valence to words/emojis reflecting the severity of different weather conditions. That is, in a similar manner to the ranking of words/emojis by their negative/positive sentiment, we used CIDER to rank them by the severity of the weather condition to which they refer, essentially detecting how language use changes as the weather changes. The steps below demonstrate how this training was performed to create a valence dictionary for temperature:

1. Filtered weather tweets were sorted by their associated temperature z-score.
2. Words and emojis in tweets with the top 1% of temperature z-scores were tagged with the label “top1”, tweets in the top 1–2% were tagged with “top2”, and tweets in the top 2% to 3% were tagged with “top3”.
3. Similarly, the bottom percentiles were tagged with “low1”, “low2”, and “low3”. The tags were chosen because they did not appear in the original dataset before being appended to the tweet text.
4. CIDER was then trained on the full set of tweets (tagged and untagged), using the following seed word sets: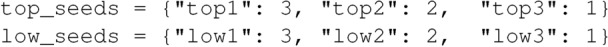 Stronger weights were assigned based on the extremity of the weather condition they are associated with. This weighting scheme allowed us to generate a tailored lexical spectrum that quantifies how words are associated with the intensity of different weather conditions.

This process was then separately repeated for precipitation, wind speed, humidity, and pressure, creating 5 distinct weather intensity classifiers with their associated lexicons. This novel approach allows us to generate weather-specific lexicons that capture the severity of each condition as expressed in language, enabling a more detailed and context-sensitive analysis of public attitudes as a result of the weather. The Python code outlining this process has been made publically available in our GitHub repository which is referenced in the “Data availability” section.

## Results

CIDER was used to create corpus-specific word/emoji scoring dimensions for positive/negative sentiment and the severity of five weather conditions (temperature, precipitation, humidity, wind and barometric pressure). These constructed scales were used to explore the different scores assigned to words/emojis, sentiment relationships with individual weather types, sentiment relationships across multiple weather conditions, and regional variations.

### Linguistic variation by weather condition

**Fig. 1:**
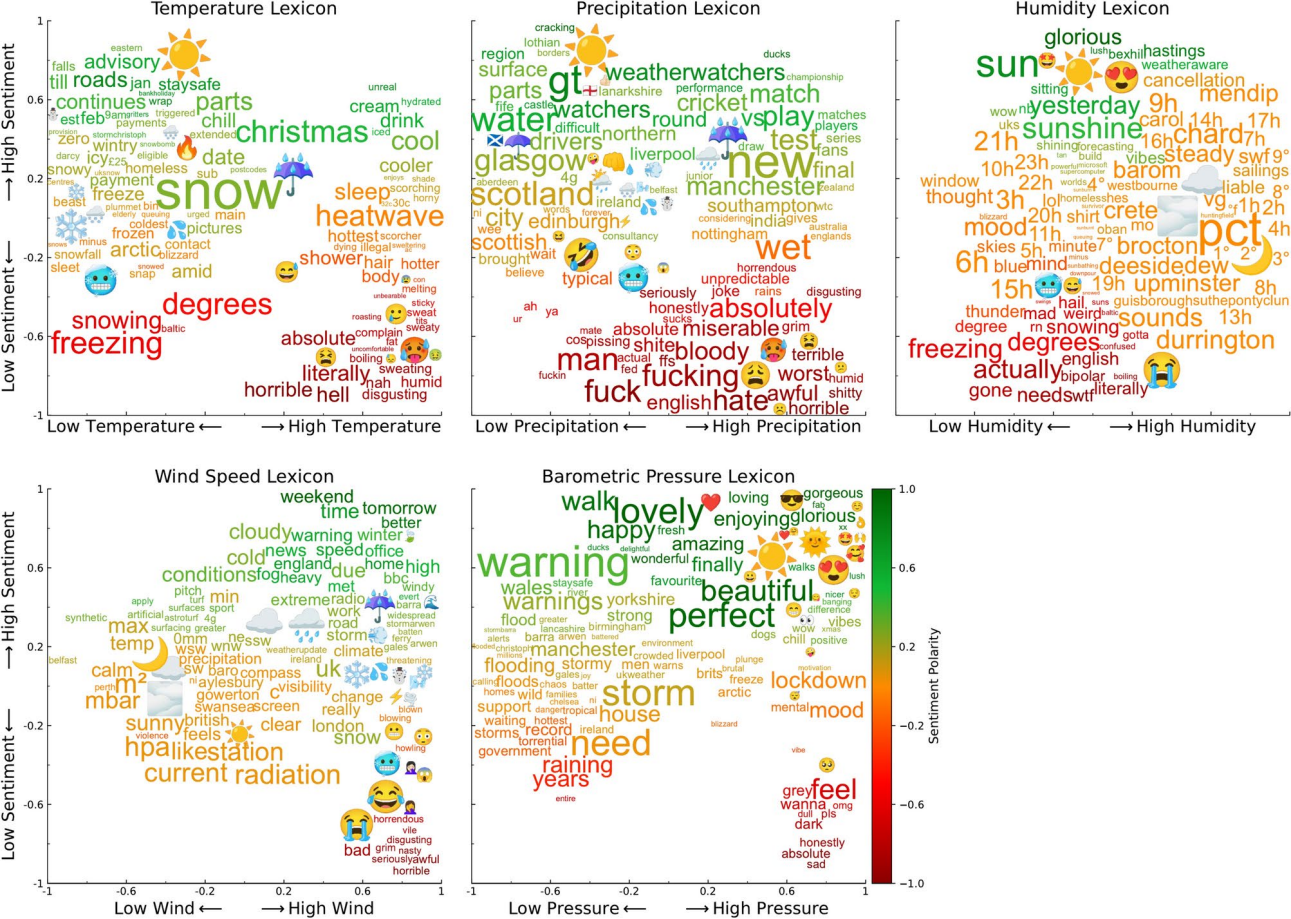
Positive/negative sentiment and weather severity associated with different words/emojis. Plots show variation of language in tweets as a result of temperature, precipitation, humidity, wind speed, and barometric pressure fluctuations, compared to sentiment fluctuations. Spectra created using CIDER^47^.

Figure 1 shows scatter plots of words/emojis scored on these dimensions, to see how language use changes in different weather conditions and sentiment polarities. The y-axis shows the learned word-level sentiment and the x-axis shows the word-level scores along the five weather scales. For example, the top left plot shows words scored by sentiment and temperature where the word “freezing” indicates low sentiment (negative) and low temperature, and “hydrated” indicates high sentiment (positive) and high temperature.

The plots in Fig. 1 show many expected associations e.g. “sweating” associated with high temperature, “wet” with high precipitation, positive emojis with positive sentiment, and so on. This provides a common-sense confirmation that CIDER polarities are meaningful. There are also some less intuitive findings, such as the small clusters found corresponding to extreme weather conditions and very negative sentiment: low sentiment/low temperature “baltic”; low sentiment/high wind “grim” and low sentiment/high pressure “sad”.

### Sentiment relationships with individual weather types

**Fig. 2:**
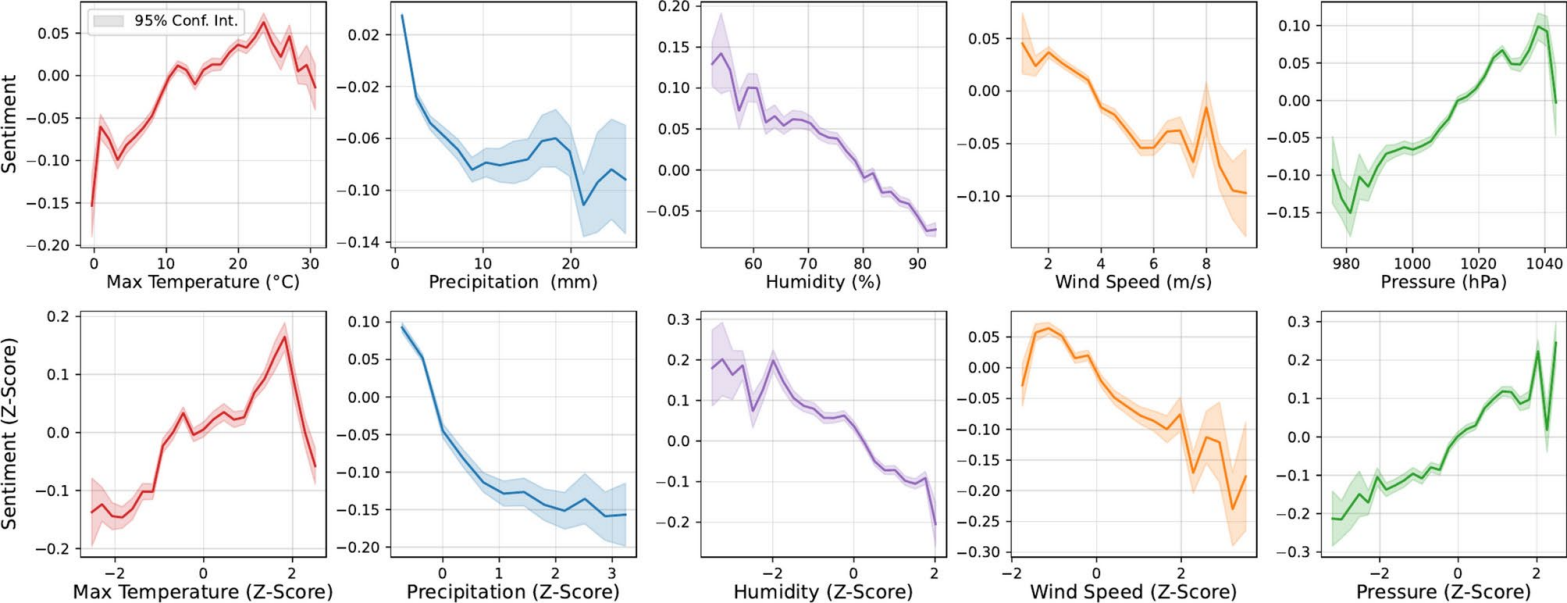
Change in sentiment at different weather conditions. Plots show sentiment Z-scores against weather conditions for every tweet in the corpus, calculated using historical gridded weather data^46^.

The sentiment and weather dimensions constructed with CIDER allows exploration of relationships between tweet-level sentiment and each weather type. Figure 2 shows the relationships between sentiment and physical weather variables. In the top row, sentiment is plotted against raw physical measurements, e.g. the average sentiment of all tweets made at 21 °C. In the bottom row, sentiment z-scores are plotted against the weather z-scores, e.g. the average sentiment z-scores of all tweets made at two standard deviations above the average temperature. Each weather condition was independently divided into 30 evenly spaced bins, and only bins containing at least 0.1% of all tweets were plotted.

The z-score transformation smooths the curves and emphasises trends that are less visible in the absolute plots, particularly by accounting for regional variations in weather. For instance, for temperature, $z=2$ corresponds to °C in Glasgow and 27.92 °C in London. Similar regional variations were apparent for precipitation, with $z=2$ reflecting 15.77 mm of rain in Glasgow and 9.4 mm of rain in London. Use of z-scores allows reliable comparisons of sentiment relationships that control for variation in physical and sentiment responses across regions.

The z-score plots in Fig. 2 reveal clear sentiment patterns across weather types. For temperature, sentiment rises significantly when temperatures exceed $z \simeq 1$, peaks around $z \simeq 1.5$, and declines sharply for $z>2,$ which is less evident in the absolute temperature plots due to regional differences. Sentiment falls rapidly with precipitation $z > 0$, saturating at around $z > 1$ and decreasing more slowly, with z-scores smoothing variability and highlighting a stronger negative relationship. The z-score transformation has minimal impact on pressure and wind speed, but it sharpens the decline in sentiment on very humid days, better capturing extreme conditions compared to the absolute values.

Note also that the z-transformation increases the range of sentiment scores significantly for all variables apart from temperature e.g. sentiment versus absolute humidity varies between around − 0.05 and 0.2, while z-transformed sentiment versus z-transformed humidity varies between around $-0.2$ and 0.3. Using absolute values for weather, there is an averaging effect on sentiment scores whereby the same weather condition drives more/less extreme sentiment in different geographic areas, depending on how unusual it is in the context of prevailing local weather conditions. Using z-scores for weather conditions measures sentiment against the deviation from prevailing conditions, rather than the absolute level; hence more extreme sentiment scores arise in locally unusual weather conditions.

### Sentiment relationships with combined weather conditions

The identified weather conditions do not occur independently of each other, for instance, high humidity is less common during periods of extreme wind, and high pressure often co-occurs with high temperatures. Combinations of conditions like high winds at the same time as high precipitation, i.e. a storm, are likely to elicit a unique response. Therefore, we next investigate how the sentiment of tweets changes in response to combinations of these conditions. To enable a visual inspection of the variation, a pairwise analysis is carried out, where a hexbin heatmap is plotted with the colour dictating the average sentiment of the tweets at that pair of weather conditions in the UK.

**Fig. 3:**
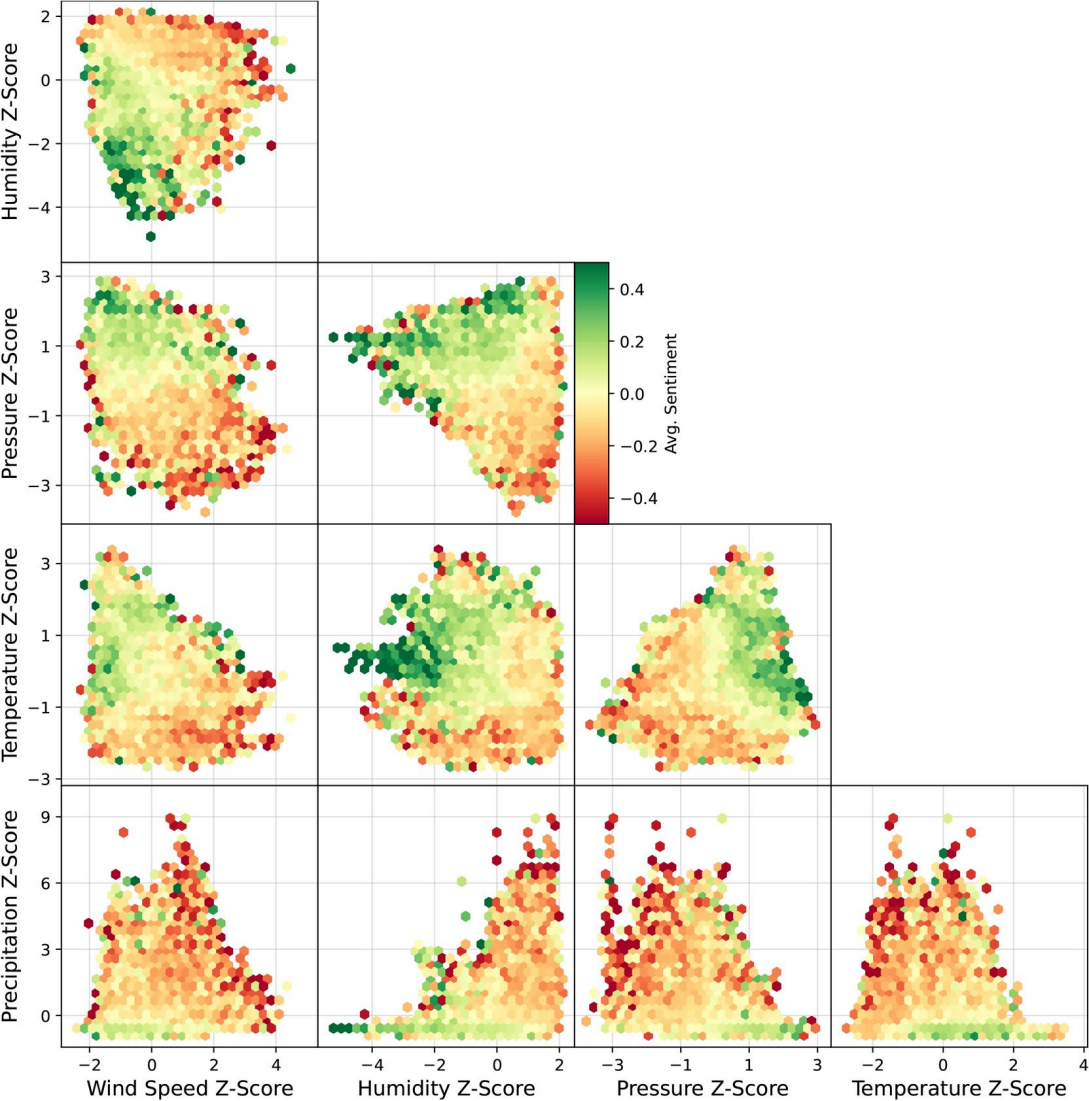
Hexbin plot comparing the average sentiment of tweets at different combinations of weather conditions.

Figure 3 shows the varying sentiment across combinations of weather conditions from tweets in the UK. Where the bin has fewer than 5 tweets, it has been omitted due to lack of volume. This is due to not all combinations of weather occurring in the data, for example, there are no days that are both high temperature (z-score > 2) and high wind (z-score > 3). For conditions that do co-occur, the response of sentiment is non-trivial. For example, at the locally average temperature, $z=0$, sentiment and humidity (third row, second column) are in an inverse relationship - high sentiment at low humidity and vice versa. At low temperatures $z < -1$, sentiment is negative for any value of humidity. On the other hand, at moderately high temperature $1<z<2$ sentiment is high regardless of humidity. At the highest observed temperatures $z>2$, there isn’t a great range of humidity observed, but sentiment is negative regardless. In general, the preferred weather of UK Twitter is low-humidity, moderately high temperature, low to no wind and high pressure. The worst weather is low temperature and pressure with high wind, precipitation and humidity i.e. storms.

Together Figs. 2 and 3 demonstrate that emotional response to weather is not straightforward - it is non-linear, multivariate, and depends on average local conditions.

### Regional sentiment variations

**Fig. 4:**
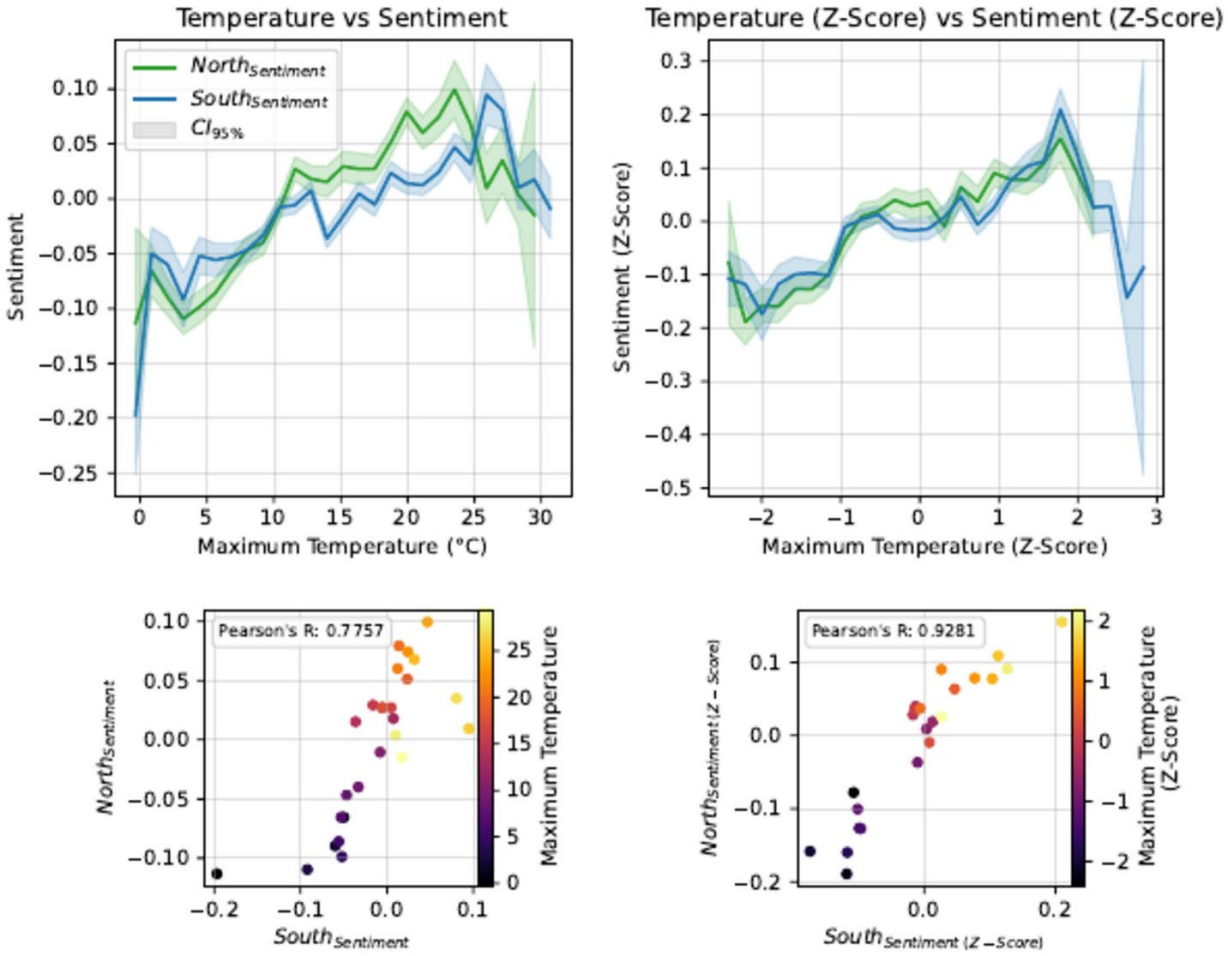
North vs South sentiment responses to temperature in the UK. Conditions and Sentiment have been normalised by z-scores on the right-hand side.

Next, an investigation into the regional variation of weather perception was carried out. For this, tweets were split into datasets for the North and South of the United Kingdom. To determine whether a tweet was from the North or South, tweets were assigned to a NUTS1 geographical region. If at least 50% of a tweet’s geolocated polygon (obtained from Section “Methodology”) was found within a single NUTS1 region, it was assigned to the corresponding NUTS1 region. This filtering method removed tweets that were broadly identified (e.g. “England” or “UK”) and geographically ambiguous tweets. Tweets from the regions “Scotland”, “North East England”, “North West England”, and “Yorkshire and The Humber” were assigned to the North, while tweets from “London”, “South East England”, “South West England”, and “East of England” were assigned to the South. Due to the significant north-south length of the UK, there are notable differences in average winter and summer daily maximum temperatures between these regions. For instance, the average summer daily maximum temperature in the North is 18.71 °C, compared to 20.88 °C in the South. In winter, the average daily maximum temperature is 5.65 °C in the North and 8.56 °C in the South, as calculated from Section “Weather condition data collection” data. The North/South axis also represents the primary cultural and linguistic divide in the UK^33^.

Focusing on maximum daily temperature, Fig. 4 presents the same information as Fig. 2, but with separate plots for the North and South of the United Kingdom. Similar to Fig. 2, data points were only plotted if the bin contained at least 0.1% of all tweets. The absolute plot (top left of Fig. 4) shows that for the same absolute temperature, the sentiment in the North is more intense (higher or lower) than the response in the South, and the positive response to warm weather peaks earlier in the North.

Plotting the z-scores (top right of Fig. 4) brings the two curves into close agreement. Note that the agreement is worse if we only normalise temperature but not sentiment and vice versa. When accounting for local temperature baselines (with the South being warmer) and local sentiment baselines (with the North expressing more intense sentiments), we find that both regions exhibit the same response to temperature. This response includes negative sentiment at low temperatures, approximately linearly increasing up to a peak at around $z=1.5$, then a fairly rapid decline at higher temperatures. This suggests that the optimal temperature in the UK is 1.5 standard deviations above the local average, regardless of what that average is.

The bottom two plots are a statistical check of the visual comparison. Plotting the green curve against the blue one will give a straight line if the two are identical. There is a significant increase in correlation, from 0.776 ($P<0.001$) to 0.928 ($P<0.001$). Similarly, applying z-score normalisation to other weather variables also resulted in increased correlations between the North and South regions, as illustrated in Supplementary Fig. S1 online.

## Discussion

Our analysis confirms a consistent response in social media sentiment to weather, aligning with previous findings. However, our work extends this understanding by examining how baseline meteorological conditions and regional differences in climate and dialect influence this response.

Firstly, we demonstrate that the concept of a “social Beaufort scale”^24^, which was initially designed to estimate wind speeds from social media, can be automated and extended to other weather conditions, such as temperature, precipitation, and humidity by applying CIDER and a simple algorithm for selecting seed words. Figure 1 illustrates these results, which also shows word-level sentiment. This approach could be applied in reverse, to infer weather conditions from tweet text. This is becoming increasingly important given the observations of Moore et al.^22^ and others on the decreasing notability of extreme weather. Even if the volume of social media is no longer a useful barometer of weather impact, analysis of the text can still be used to infer both public mood and the weather conditions themselves.

We find that trends in sentiment are more clearly emphasised when local weather baselines are taken into account, as shown in Fig. 2. This is particularly important for temperature in the UK, which spans approximately 10 degrees of latitude, resulting in significantly colder conditions in the north compared to the south. Without considering these local baselines, the response of public mood to extreme temperatures remains obscured. When we account for local baselines, we observe a significant decrease in sentiment as temperatures rise $\sim 2 \sigma$ above normal. There may be a temporal as well as a spatial component, as people’s baseline expectations change over time with a warming climate. Although we currently lack a sufficiently long time series to explore this fully, future studies understanding changing responses over time are crucial for social media analysis to effectively contribute to understanding public perception and response to weather in a changing climate.

We also find that public mood is influenced in a non-linear way by combinations of weather conditions, as shown in Fig. 3. For example, a relatively high temperature ($\sim z=1$) can be associated with both positive or negative sentiment depending on humidity levels. Climate change affects all aspects of the climate, so understanding or predicting changes in public mood requires multi-dimensional analyses. In general, weather conditions that cause particularly strong negative or positive reactions are not surprising e.g. high precipitation and high wind causing negative sentiment. However, there are intriguing, less obvious effects, such as unusually high pressure being associated with negative sentiment. This is also evident in the high-pressure cluster in Fig. 1, where words like “grey, dark, vibe, feel, sad, ...” are prominent.

Finally, our most significant finding, illustrated in Fig. 4, demonstrates the importance of both cultural and weather baselines. The north-south axis divides the UK into distinct cultural and climate regions. The north is colder and expresses sentiment more strongly on social media than the south^51^. This results in the ideal temperature peak occurring at lower temperatures in the north than in the south, suggesting a lower temperature baseline. However, the sentiment at colder temperatures is *more* negative in the north. When we account for local conditions and variations in sentiment intensity, we find that the two curves align, suggesting a universal response to weather once both baselines are considered.

This has significant implications for all analyses of social media responses to weather. Most countries and regions have a diversity of climates and languages, meaning that average expectations may not accurately reflect the experiences of any specific group. Methods like CIDER, which are flexible enough to account for local and contextual variations in communication, are crucial for this type of analysis to be useful to forecasters. For example, the move towards impact-based forecasting^52^ emphasises the social dimensions of weather, with social media analysis having been used to validate these predictions^9,53^. Failing to account for variations in local dialects risks under- or overestimating weather impacts. Many academics and forecasting professionals aim to evaluate and improve risk communication strategies^54^. Understanding local dialects can help not only in evaluating the social media response to such messaging but also in creating bespoke messages for different communities.

There are limitations to our study however, for instance, CIDER, like many lexicon-based sentiment analysis techniques, is unable to detect some nuanced expressions such as irony or sarcasm (e.g., “The weather is just great” in reference to bad weather). While lexicon approaches offer transparency and simplicity, they lack the depth of more advanced NLP methods. Additionally, some of our analysis assumes sentiment changes are consistent over time, but evolving public attitudes towards climate and weather introduce temporal variability^15^. Finally, biases in social media platform usage and demographic representation may limit the generalisability of our findings.

This paper builds on studies like Baylis et al.^12^ but introduces key advancements. We use context-dependent sentiment analysis, examine a wider range of weather conditions, and develop a weather lexicon to link specific words to conditions. We also analyse combinations of weather factors and provide a north-south UK comparison, accounting for cultural and climate differences. These insights offer a more comprehensive understanding of how weather affects public mood.

Approaches to studying weather on social media using volume and general-purpose NLP methods have generated numerous insights. Our study demonstrates that accounting for weather and linguistic baselines is crucial for achieving a more accurate and nuanced analysis. We also recognise that in a changing climate and an ever-evolving social media landscape, flexible and transparent methods are essential to ensure that the information available to academics, forecasters, and weather professionals remains relevant and useful. We believe the methods and results presented in this paper represent a significant advancement beyond previous approaches to social media analysis of weather.

## Supplementary Information

Supplementary Information.

## Data Availability

The datasets and code used in this study are available from the GitHub repository https://github.com/jcy204/LanguageOfWeather/ in compliance with Twitter’s data sharing guidelines.
