# Supplementary material for: The linguistic and emotional effects of weather on UK social media users
Source: Sci Rep. 2025 Mar 7;15:8009. doi: 10.1038/s41598-024-82384-w (PMC11889188; doi:10.1038/s41598-024-82384-w)
Supplement: Supplementary file 1 — Supplementary Information. [file 41598_2024_82384_MOESM1_ESM.pdf]

Supplementary Figure S1:
Extended Regional Sentiment Variations

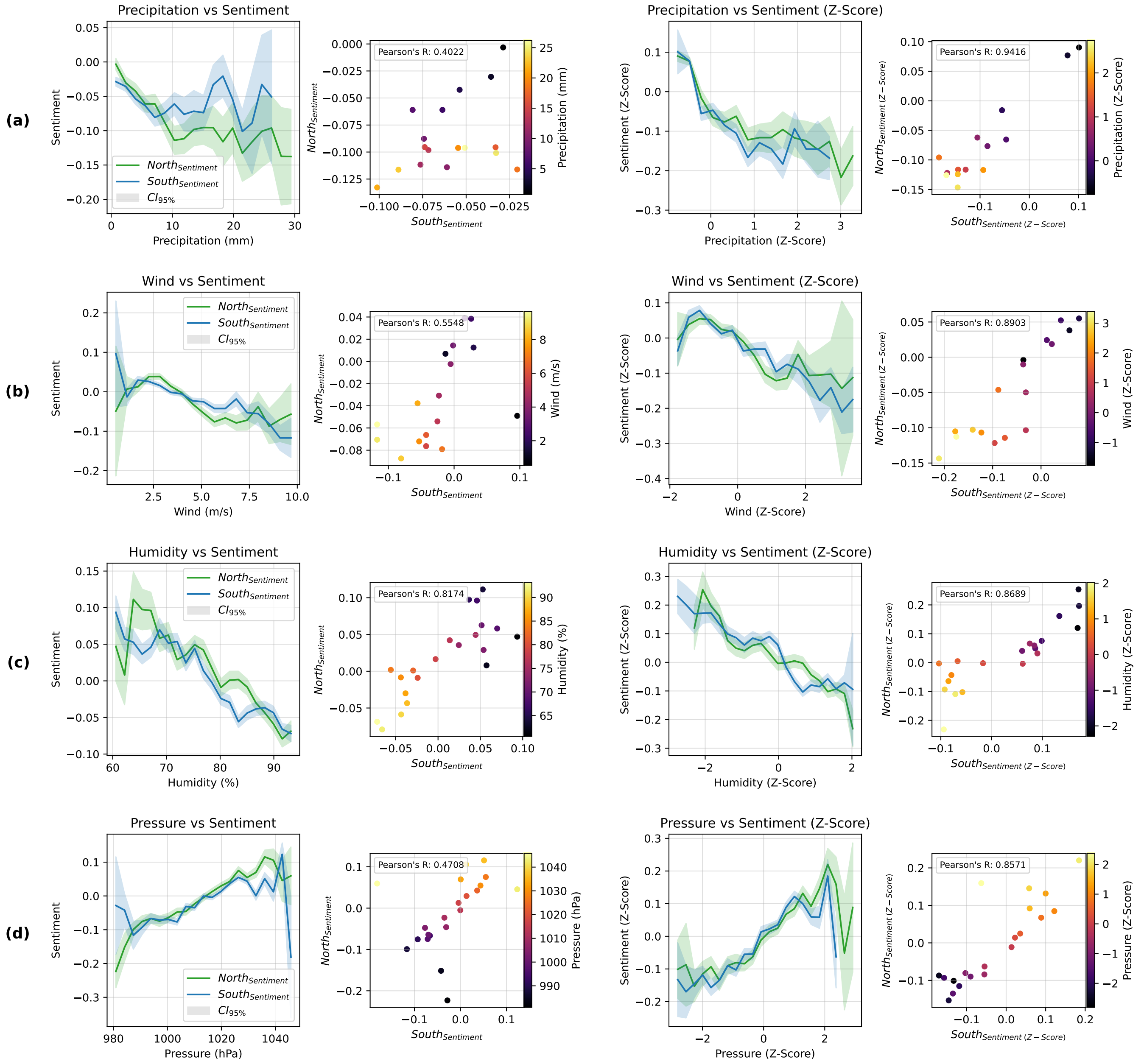

Figure S1: North vs South sentiment responses to precipitation, wind, humidity, and pressure variations in the UK. Conditions and sentiment have been normalised by z-scores on the right-hand side.
